# The Control of Typhoid Fever in Vietnam

**DOI:** 10.4269/ajtmh.18-0035

**Published:** 2018-07-25

**Authors:** Tran Vu Thieu Nga, Pham Thanh Duy, Nguyen Phu Huong Lan, Nguyen Van Vinh Chau, Stephen Baker

**Affiliations:** 1 Wellcome Trust Major Overseas Programme, Hospital for Tropical Diseases, Oxford University Clinical Research Unit, Ho Chi Minh City, Vietnam;; 2 Department of Medicine, University of Cambridge, Cambridge, United Kingdom;; 3 Centre for Tropical Medicine and Global Health, Nuffield Department of Clinical Medicine, Oxford University, Oxford, United Kingdom

## Abstract

Typhoid fever, caused by *Salmonella enterica* serovar Typhi (*S*. Typhi), is a diminishing public health problem in Vietnam, and this process may represent a prototype for typhoid elimination in Asia. Here, we review typhoid epidemiology in Vietnam over 20 years and assess the potential drivers associated with typhoid reduction. In the 1990s, multidrug resistant *S*. Typhi were highly prevalent in a sentinel hospital in southern Vietnam. A national typhoid incidence rate of 14.7/100,000 population per year was estimated around the new millennium. The Vietnamese government recognized the public health issue of typhoid in the 1990s and initiated vaccine campaigns to protect the most vulnerable members of the population. At their peak, these campaigns immunized approximately 1,200,000 children in 35 provinces. Concurrently, Vietnam experienced unprecedented economic development from 1998 to 2014, with the gross national income per capita increasing from $360 to $1,890 over this period. More recent typhoid incidence data are not available, but surveillance suggests that the current disease burden is negligible. This trajectory can be considered a major public health success. However, a paucity of systematic data makes it difficult to disaggregate the roles of immunization and water, sanitation, and hygiene (WASH) interventions in typhoid reduction in Vietnam. Given the limitations of typhoid vaccines, we surmise the practical elimination of typhoid was largely driven by economic development and improvement in general population living standards. Better designed WASH intervention studies with clinical endpoints and systematic incidence data are essential to glean a greater understanding of contextual factors that impact typhoid incidence reduction.

## INTRODUCTION

Typhoid fever, the disease caused by *Salmonella enterica* serovar Typhi (*S*. Typhi), is a diminishing public health problem in Vietnam.^1^ However, the disease remains an ongoing public health issue in other parts of South and Southeast Asia,^2–4^ and an enhanced understanding of disease estimates and the influence of antimicrobial resistance (AMR) on disease presentation is needed to better control this disease across the region. Furthermore, insights into the trends of typhoid and factors that directly impinge on disease incidence are important for allocating resources for reducing the burden of disease.^5^ Currently, Vietnam represents an exemplar Asian country that has all but eliminated this once common infection, and there is much to be learnt from the reduction of typhoid in Vietnam. However, how reduction in typhoid was precisely achieved is unclear, and providing a roadmap for typhoid reduction in similar settings is largely dependent on good historical quantitative data.

### Typhoid fever incidence in Vietnam.

**Figure 1.**
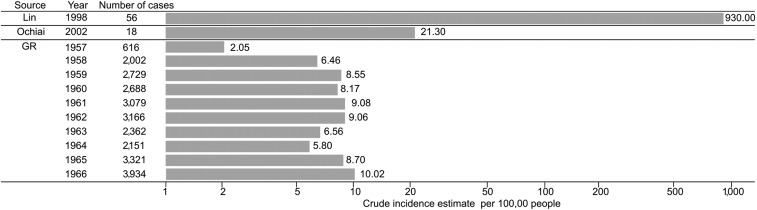
Historic crude estimates of typhoid fever incidence in Vietnam. Histogram showing the estimated crude incidences (on a log scale) of typhoid fever in Vietnam from government records (GR) and subnational incidence estimates available from Ochiai et al.^9^ (aggregated estimated from 2002 to 2004, with 2003 as the midpoint from Hue province in people aged 5–18 years) and Lin et al.^13^ (aggregated estimated from 1997 to 2000, with 1998 as the midpoint from Dong Thap province).

Typhoid fever has likely been endemic in Vietnam for some time, although historical incidence data for this common cause of febrile disease from across Southeast Asia before the reunification of Vietnam in 1975 is scarce. Notified typhoid fever cases reported to the pre-reunification government of South Vietnam showed a generally increasing trend from 2.05 cases per 100,000 people annually in 1957 to 10.02 cases per 100,000 people annually in 1966 (Figure 1).^6^ Health care provisions, and water and sanitation infrastructure in South Vietnam during this time period were generally poor, which likely contributed to the increasing rates of typhoid fever and other infectious disease during this period, which were frequently observed in military personnel returning back to the United States.^7^ By contrast, a report suggests that the pre-reunification government in North Vietnam prioritized health care access and began mass vaccination campaigns against typhoid fever and other communicable diseases as early as 1954, although reliable incidence data from North Vietnam during this period are not available.^8^

**Figure 2.**
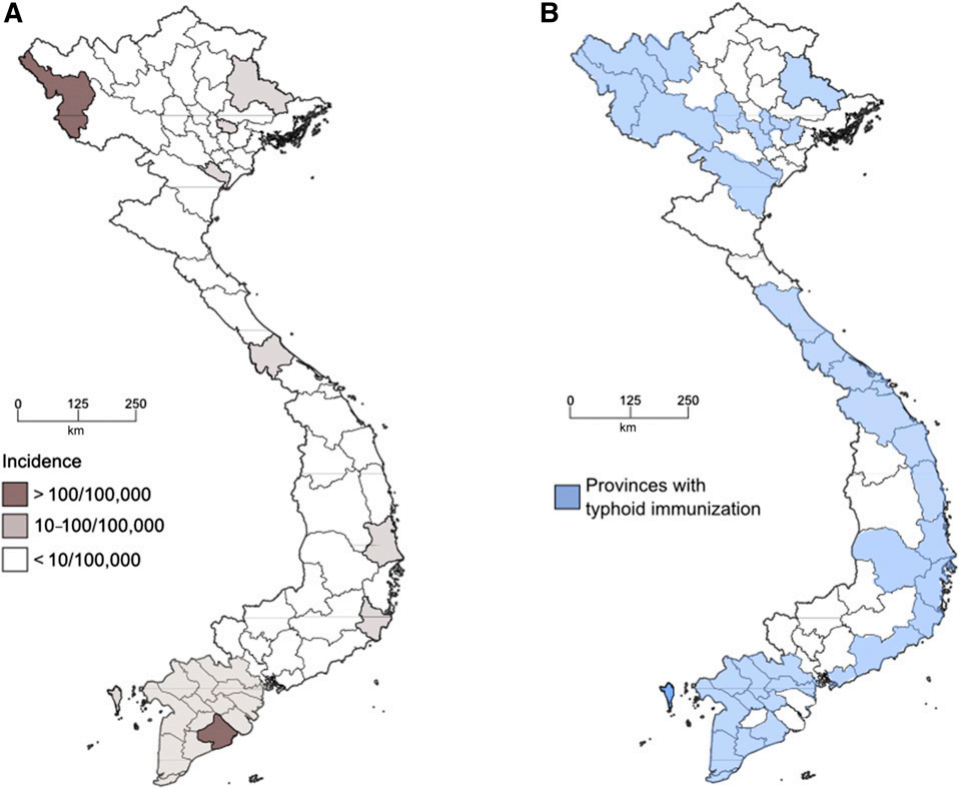
Map of Vietnam showing estimated disease incidences and provinces implemented Vi immunization. (**A**) North orientated map of Vietnam showing the estimated incidence of typhoid fever in Vietnam from government data between 1999 and 2003. Provinces with high, medium, and low incidence are highlighted by shading (see key). (**B**) North orientated map of Vietnam showing the 35 provinces in Vietnam in 2005 that were incorporated into the national typhoid Vi immunization campaign; blue shading (see key). Maps are reproduced from Cuong N. Typhoid Vaccine Used in Vietnam and its Impact. In: Consultation on Typhoid Vaccine Introduction and Typhoid Surveillance.^12^

The best and most accurate recent estimates of typhoid incidence in Vietnam were calculated during the International Vaccine Institute’s Diseases of the Most Impoverished (DOMI) program, which was conducted between 1999 and 2003.^9^ The annual incidence of typhoid fever in Hue, in central Vietnam, in 2002–2004 was estimated to be 21.3/100,000 person years and 24.2/100,000 person years in children aged 5–15 in years (Figure 1). This program went on to conduct various epidemiological investigations and vaccine studies in the same location.^10,11^ In addition, the National Institute of Epidemiology (NIHE) in Hanoi conducted further nationwide surveillance around the same period of time as the DOMI study. The average number of typhoid cases in Vietnam across the country in all ages was estimated to be 11,696, corresponding with an average national incidence rate of 14.7/100,000 population per year.^12^ During this period (1999–2003) two of the 63 provinces of Vietnam (Soc Trang in the south and Dien Bien in the north) were estimated to have particularly high incidences (> 100/100,000 population per year) (Figure 2); a further 18 were estimated to have a medium incidence (> 10 < 100/100,000 population per year). The propensity of the disease was understood to arise in children, with an estimated incidence of 36.6/100,000 population aged < 15 years per year.^12^ Lastly, in 1998, Lin and others.^13^ estimated a population incidence of 198/100,000 population in the Dong Thap province in the Mekong Delta, equating with a crude incidence of 930/100,000 people (Figure 1).

### Typhoid trends in Ho Chi Minh City (HCMC).

**Figure 3.**
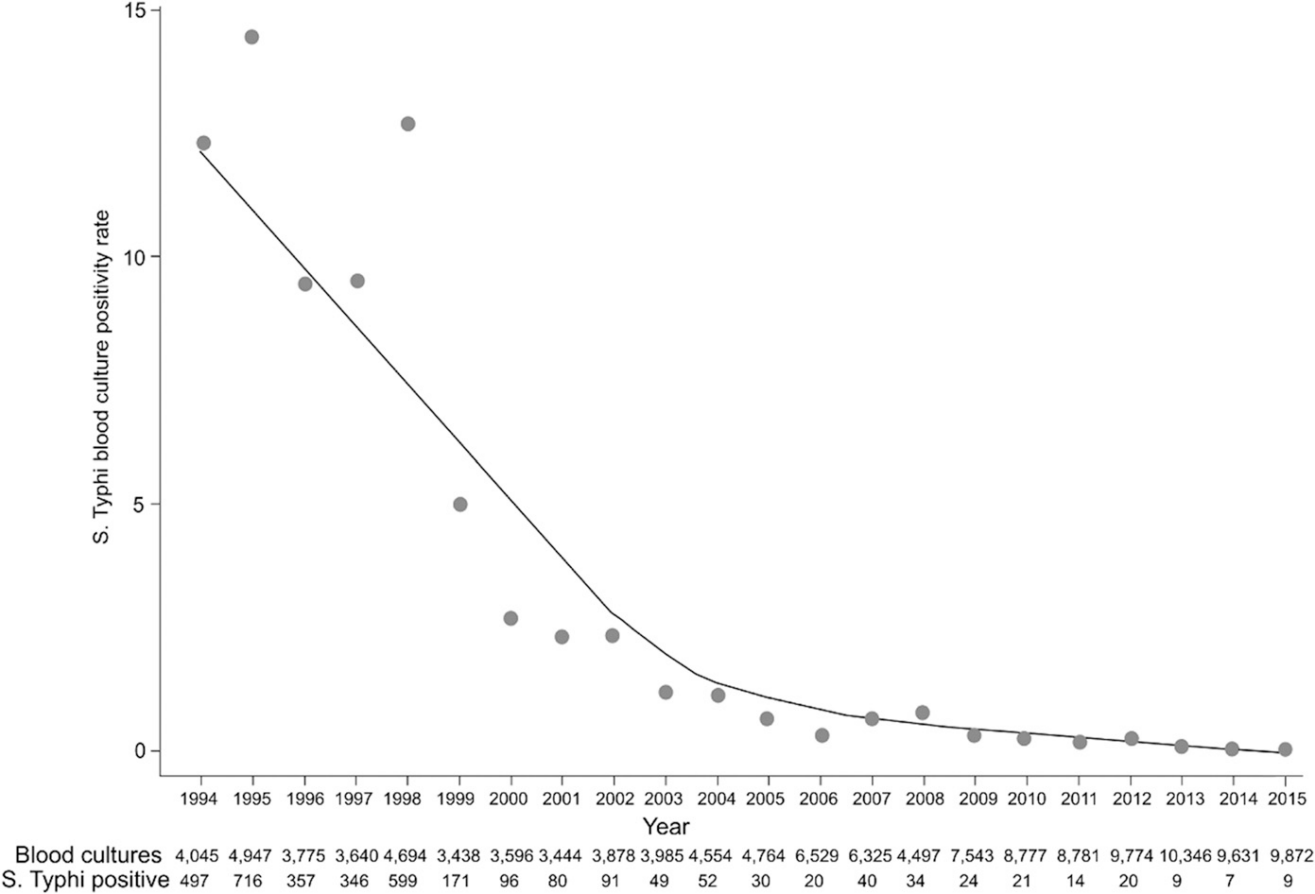
The decline in *Salmonella* Typhi–positive blood cultures in a sentinel infectious disease hospital in Ho Chi Minh City (HCMC). Plot showing the proportion of total blood cultures taken from which *Salmonella* Typhi was isolated between 1993 and 2015 at the Hospital for Tropical Diseases in HCMC, with a locally weighted scatterplot smoothing curve. The total number of blood cultures taken and the number from which *Salmonella* Typhi were isolated are shown at the base of the figure.

Routine blood culture data from the Hospital for Tropical Diseases (HTD) in HCMC in the south of Vietnam between 1994 and 2015 highlights a major reduction in the prevalence (and absolute number) of positive blood cultures for *S*. Typhi over time (Figure 3).^1^ Hospital for Tropical Diseases is a sentinel infectious disease hospital that serves as a primary and secondary facility for the surrounding local population in HCMC and a tertiary referral center for 17 provinces in the south of the country, and, therefore, has a catchment population of approximately 40 million people. The highest rates of positive blood cultures for *S*. Typhi at HTD were recorded in 1995 and 1998, when the proportion of positive blood cultures for *S*. Typhi was 14.5% and 12.8% (of all blood cultures taken), respectively. In the late 1990s, this figure began to show an annual decline; 5% *S*. Typhi blood culture positivity rate of all blood cultures taken in 1999. After the turn of the millennium, the number of culture positive cases of typhoid fever at HTD continued to decrease annually, with the prevalence of *S*. Typhi–positive blood cultures not rising higher than 1% from 2005 onward. Therefore, in the absence of contemporary (and accurate incidence) data, if we extrapolate these trends we can surmise that presently the incidence of enteric fever in Vietnam is probably exceptionally low (< 10/100,000 population per year), and there has been a remarkable and sustained decline in the prevalence of *S*. Typhi–positive blood cultures in HTD and other health care facilities across the country.

In a pattern similar to those observed in parts of sub-Saharan Africa (but not in the same magnitude), there has been a replacement of “classical” community-acquired pathogens in bloodstream infections (such as *S*. Typhi) with those more commonly associated with HIV infection and the current international epidemic of AMR bacteria.^14^ Specifically, assorted fungal pathogens, multidrug-resistant (MDR) non-*Salmonella* Gram-negative bacteria, and non-typhoidal *Salmonella* now dominate the bloodstream infection landscape in Vietnam.^1,15,16^ Paratyphoid fever, which is associated with the various pathovars of *Salmonella* Paratyphi (*S*. Paratyphi A, B, and C), has been reported to be increasing in prevalence in parts of Asia.^17^ This surge is specifically associated with *S*. Paratyphi A; however, the isolation of this organism is rare in Vietnam (and across Southeast Asia) and is generally limited to extended sporadic outbreaks, as recently observed in neighboring Cambodia.^18^ A study conducted at HTD cultured less than 7 *S*. Paratyphi A isolates per year between 1998 and 2008, this subsequently declined to zero from 2008.^1^ However, in 1990, Global Burden of Disease estimated incidence of paratyphoid to be 81/100,000 population; this was estimated to be 40/100,000 population in 2016.^19^

### Antimicrobial susceptibility.

Traditionally, Vietnam has been a global hotspot for multi-drug resistant (MDR) *S*. Typhi, which is defined as resistance against the first-line antimicrobials, ampicillin, chloramphenicol, and trimethoprim-sulphate.^20^ These latter day first-line regimes were commonly prescribed in the community and in health care settings for the treatment of typhoid fever, and many non-specific febrile diseases, in Vietnam in the 1980s and early 1990s. The first notable spike of MDR *S*. Typhi in Vietnam arose in the early 1990s, correlating with a major peak in *S*. Typhi–positive blood cultures at HTD in HCMC.^21^ This MDR phenotype in *S*. Typhi was associated with an incH1 plasmid backbone,^22^ which has been consistently identified within *S*. Typhi isolated in Vietnam and coupled with organisms belonging to a specific phylogenetic group known as haplotype 58,^23^ now designated genotype 4.3.1.^24^ These organisms, with this same MDR phenotype, were described as still circulating in high numbers in the Mekong Delta region, some 150 km away from HCMC, in 2004 and 2005.^25^

*Salmonella enterica* serovar Typhi belonging to genotype 4.3.1 are also commonly associated with a mutation (S83F) in the DNA gyrase gene *gyrA*, catalyzing resistance against naladixic acid and reduced susceptibility against the second-generation fluoroquinolones, ciprofloxacin, and ofloxacin.^26,27^ Variants with this specific *gyrA* mutation began to emerge in Vietnam in the early 1990s,^28^ shortly after the introduction of quinolones for the treatment of non-specific febrile diseases when the first-line treatments became less effective at inducing defervescence. The secondary peak in *S*. Typhi cases in routine blood culture data from HTD was associated with the emergence of organisms exhibiting resistance against quinolones and reduced susceptibility against fluoroquinolones.^1,29^ These organisms, specifically genotype 4.3.1 *S*. Typhi with an S83F mutation in *gyrA*, have since become the most prevalent variant in Vietnam,^25^ reflecting the pattern of the molecular epidemiology of *S*. Typhi across much of Asia.^27^ Current information regarding the AMR profiles of the extant *S*. Typhi population in Vietnam are limited, but our unpublished data suggests that MDR strains have all but disappeared in the southern part of the country and *S*. Typhi with an S83F and reduced susceptibility to fluoroquinolones continue to circulate. Notably, despite the sustained use and availability of fluoroquinolones in Vietnam for various bacterial infections, ciprofloxacin- and ofloxacin-resistant *S*. Typhi has yet to emerge, although it has been observed elsewhere in Asia.^30^

### Vaccination campaigns.

The Vietnamese government recognized the public health issue of typhoid fever in the 1990s and initiated several vaccine campaigns with internationally manufactured vaccines in an attempt to protect the most vulnerable groups within the population, most commonly children.^31^ Furthermore, NIHE in Hanoi was a pioneer in instigating locally manufactured Vi Polysaccharide vaccine and distributing it as a control measure through the public health network. However, despite a Vi conjugate vaccine and a new oral attenuated typhoid vaccine being trialed in Vietnam for the first time, these were never introduced as public health interventions.^32,33^ All the national typhoid Vi Polysaccharide vaccine programs conducted between 1997 and 2012 were executed as school-based campaigns, immunizing children between the ages of 3 and 10 years. Between 1997 and 2003, the Vietnamese government immunized more than 4,000,000 children aged 3–5 years with TYPHIM Vi polysaccharide, manufactured by Aventis Pasteur. After manufacturing their own Vi polysaccharide vaccine through the Institute of Vaccines and Medical Biologicals/Da Lat Pasteur Vaccine Company, the Vietnamese public health system administered more than 2,000,000 additional doses to children aged 5–10 years (2004–2010), and latterly children aged 3–5 years (2011–2012). At the peak of these Vi vaccine campaigns in 2005 (1,200,000 doses), children were being immunized in 35 different provinces across the country (Figure 2).^12^ The coverage rate of these campaigns were high, with > 90% of the target population receiving a Vi vaccine between 1999 and 2010. Population based data assessing the direct effect (i.e., without additional sanitation covariates) of these immunization programs on typhoid incidence are unavailable, but there was a substantial decrease in the incidence of typhoid fever across the country between 1997 and 2007, most notably in the northwest of the country and the Mekong River Delta in the South, which were covered extensively by the immunization program (Figure 2).^12^

### Contextual factors that may have influenced typhoid fever incidence.

**Figure 4.**
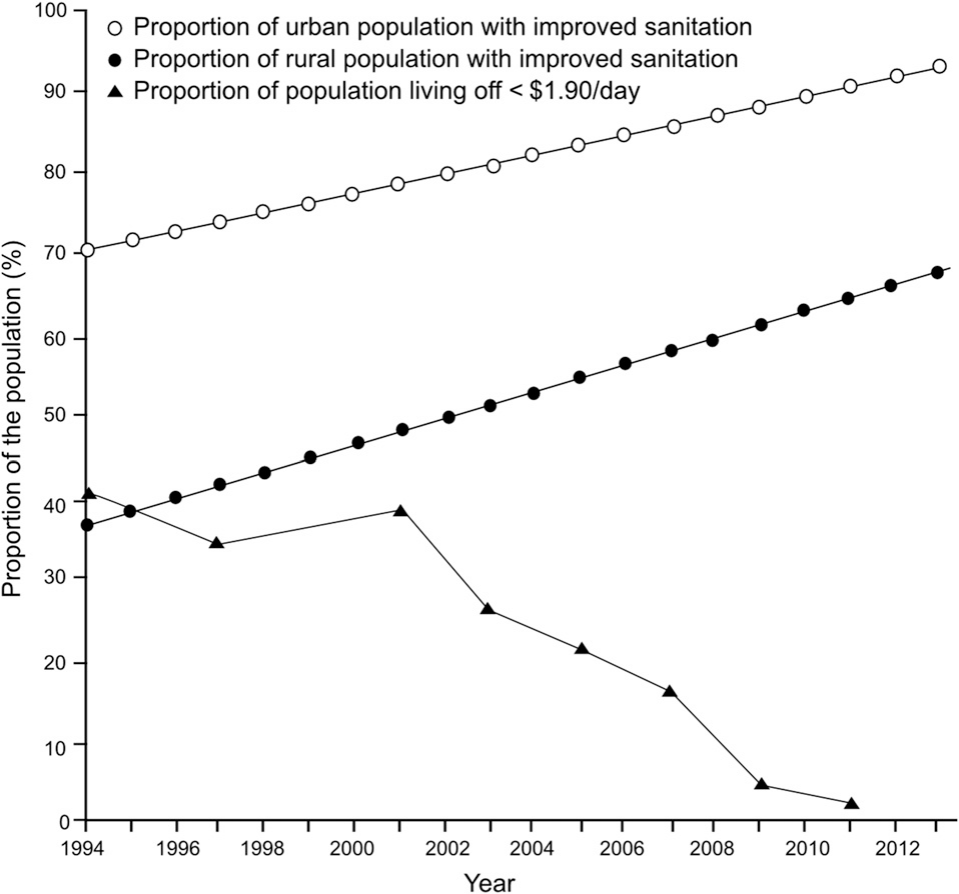
The reduction of poverty and improvements in sanitation in Vietnam. Plots showing the proportion of the Vietnamese population living on < $1.90 a day (black triangles), the proportion of the Vietnamese rural population with improved sanitation (black circles), and the proportion of the Vietnamese urban population with improved sanitation (white circles) from 1994 to 2013.

Being of low socioeconomic status is a major risk factor for contracting typhoid fever.^34^ Vietnam has been through an unprecedented period of economic development since the mid-1990s, which has had a substantial knock-on effect on the reduction of poverty and poverty-associated communicable diseases.^35^ Between 1998 and 2014 Vietnam’s gross domestic product increased from $27 billion to $86 billion, and the gross national income per capita increased from $360 to $1,890 over the same time period.^36^ These figures correlate with a reduction in national poverty (poverty headcount ratio at $1.90 a day declining from 34.8% in 1998 to 3.2% in 2012) (Figure 4) and a waning in typhoid fever incidence, but overlap with the period of the national immunization campaigns. In addition, multiple contextual factors are considered to have had an effect on the incidence of typhoid fever. Typhoid fever is associated with poor water quality, and evidence of the organism in water supplies can be measured using molecular methods.^37^ Consequently, water, sanitation, and hygiene (WASH) conditions are one of the major factors in assessing disease control. Sub-national data on water and sanitation extracted from Multiple Indicator Cluster Surveys (MICS) in Vietnam show that the fraction of the population in the southeast of Vietnam with access to improved sanitation facilities (means of excreta disposal that decrease human contact with feces) increased from only 42% in 1995 to 93.6% in 2014.^38^ Furthermore, the proportion of the southeastern population of Vietnam with improved water sources (as per MICS standards; water piped into homes or yard, public taps, standpipes, protected wells, or springs) rose from 93.6% in 2006 to a peak of 98.4% in 2011.^38^ Correspondingly, data from the World Bank shows that there was a steady increase in the proportion of the Vietnamese population with access to improved sources of drinking water between 1998 (74.5% of the population) and 2014 (96.4% of the population).^39^ Similarly, there was an increase in the proportion of the population with access to improved sanitation facilities, including flush and slab latrines, over the same period; 49.5% of the population in 1998 to 76.3% of the population in 2012 (urban/rural data shown in Figure 4).^39^

### Knowledge gaps.

All available data suggest that the trend of typhoid fever incidence began to exhibit a steep decline in Vietnam from 1999 onward. Therefore, the outstanding questions regarding the dramatic reduction in typhoid fever in Vietnam are: to what extent did immunization play a role in reducing typhoid fever? and how much influence did economic growth have on improving living standards to reduce all waterborne diseases, including typhoid fever? Given the paucity of data regarding the longitudinal incidences of typhoid fever (and other waterborne diseases) in Vietnam, disaggregating the independent effect of these differing approaches is a major challenge. Furthermore, dissimilar demographics, disease epidemiology, and disease incidence across the provinces of Vietnam make it impractical to assess the overall impact of a specific intervention in any given location. The data that are available are inconclusive and limited by disease time trends from a single tertiary referral hospital in HCMC. As a result, these data do not provide insight into regional differences in typhoid fever reduction across Vietnam or characterize the current national burden. Furthermore, there are no systematic data regarding intestinal perforation (although this has been described in the southern provinces of Vietnam^40^), other severe disease presentations,^41^ or typhoid-associated mortality. As a result, it is impossible to directly assess any trends associated with these severe outcomes.

Regardless of the precise role of any single intervention, the reduction and virtual elimination of typhoid fever in Vietnam, largely driven by strong political will,as evidenced by sanitation improvements, better health care, and immunization campaigns, should be considered a major public health success. However, this success story does not quite replicate into a blueprint in how typhoid fever should be controlled across Asia, that is, what worked in Vietnam may not work in precisely the same way in other locations. It is likely that the various vaccination campaigns that have taken place in Vietnam over the last 15 years have contributed to the observed decreasing trend in typhoid fever, but their effect is difficult to assess. However, the immunization campaigns were conducted in children in selected provinces, and these individuals received a single dose of the Vi polysaccharide vaccine. This vaccine only provides limited efficacy in the first year after immunization and there is a rapid decline in antibody titer 2 years after receiving the vaccine,^42,43^ herd protection remains variable. Therefore, we conclude that economic development, improved access to clean water supplies, better sanitation, and a reduction in poverty probably played the greatest combined role in reducing the incidence of typhoid in Vietnam. Quantifying the precise contribution of these in the reduction of typhoid incidence is again problematic, and there are multiple additional factors that should also be considered that may confound these interactions. It is, however, worth stating that *S*. Typhi appears to be profoundly sensitive to improvements in sanitation, which reduces human exposure to the organism, thus, lessening disease incidence and person-to-person transmission.

## CONCLUSION

The reduction of typhoid fever in Vietnam has been remarkable, and has been largely driven by economic development and improved living standards for the population. Immunization has probably had some impact on disease reduction, but the use of an imperfect vaccine may only provide limited respite in disease transmission without required improvements in WASH. Better designed WASH intervention studies with disease endpoints and systematic incidence data are required to glean a greater understanding of the precise contextual factors that impact on typhoid fever incidence.
